# Pathways to Suicide in Australian Farmers: A Life Chart Analysis

**DOI:** 10.3390/ijerph14040352

**Published:** 2017-03-28

**Authors:** Lisa Kunde, Kairi Kõlves, Brian Kelly, Prasuna Reddy, Diego De Leo

**Affiliations:** 1 Australian Institute for Suicide Research and Prevention, National Centre of Excellence in Suicide Prevention, World Health Organization Collaborating Centre for Research and Training in Suicide Prevention, Griffith University, Mt Gravatt Campus, Brisbane, QLD 4122, Australia; l.kunde@griffith.edu.au (L.K.); d.deleo@griffith.edu.au (D.D.L.); 2 Centre for Brain and Mental Health Research, School of Medicine and Public Health, University of Newcastle, Newcastle, NSW 2308, Australia; brian.kelly@newcastle.edu.au; 3 Faculty of Health, University of Technology Sydney, Broadway, Sydney, NSW 2007, Australia; prasuna.reddy@uts.edu.au; 4 Institute of Psychiatry, Psychology and Neuroscience, King’s College, London SE5 8AF, UK

**Keywords:** suicide, psychological autopsy, life chart, life events, farming, the Interpersonal-Psychological Theory of Suicidal Behaviour

## Abstract

Farmers have been found to be at increased risk of suicide in Australia. The Interpersonal-Psychological Theory of Suicidal Behaviour suggests that the proximal factors leading to the suicidal desire or ideation include an individual’s experiences of both perceived burdensomeness and thwarted belongingness. Suicidal desire with acquired capability to engage in lethal self-injury is predictive of suicidal behaviour. This study investigates the pathways to suicide of 18 Australian male farmers in order to understand the suicidal process and antecedents to suicide in Australian male farmers. The psychological autopsy (PA) method was used to generate life charts. Two pathways with distinct suicidal processes were identified: acute situational (romantic relationship problems and financial concerns/pending retirement) and protracted (long-term psychiatric disorder). Long working hours, interpersonal conflicts, physical illnesses and pain, alcohol abuse, access to firearms, and exposure to drought were additional common factors identified. An understanding of the interrelatedness of diverse distal and proximal risk factors on suicidal pathways in the wider environmental context for male farmers is required when developing and implementing rural suicide prevention activities.

## 1. Introduction

Male farmer suicide is an important social and public health issue in Australia [1]. Elevated suicide rates among farmers have been reported in numerous international studies, including Australia [1,2]. In Queensland, Australia, agricultural workers have been found to have the highest suicide rates compared to other occupational groups [1].

Farming has been a major contributor to Australia’s economy and cultural identity since European settlement [3]. However, the number of people residing in rural areas and/or working in agriculture has declined rapidly [3]. Over a 30 year period to 2011, the number of farmers declined by 106,200 (40%), an average of 294 fewer farmers every month over that period [3]. In recent decades, farmers have faced climatic extremes and a decline in Australia’s reliance on agriculture [3,4]. Small farmers have been selling up to large scale corporate farming operations and fewer young people are taking over family farms [4]. Consequently, farming has an ageing workforce (median age 53 years compared with 40 years in all other occupations and with almost 25% of farmers aged 65 years or over) who are more likely to continue working beyond the age most other workers retire [3].

Although farming is known to be a physically and psychologically demanding occupation, research to date has not observed a higher prevalence of mental illness among farmers compared with non-farmers [5]. Researchers have suggested a number of individual, economic, environmental and climatic stressors that may impact on farmers’ mental health, increasing the risk of suicide [4]: personality characteristics; long work hours, low income with high assets, social isolation, an ageing population, an overlap of work and family environments; poor-access to health care services [4,5,6,7]; regulatory and industry factors beyond the farmer’s control [8]; and enduring prolonged periods of climate variability [9]. There is a gap in the understanding of how and when these factors may lead farmers to suicide.

A theory that could help in understanding farmer suicide is the Interpersonal-Psychological Theory of Suicidal Behaviour (IPT) [10,11,12]. This theory posits that individuals have an inherent self-preservation instinct that is difficult to overcome. However, the proximal factors developing the desire for suicide is the presence of two interpersonal and psychological states: thwarted belongingness (feelings of social isolation or disconnection) and perceived burdensomeness (perceived lack of caring relationships, feelings of burden to others) [10,11,12]. Acquired capability is a pre-existing vulnerability or reduced fear of death and increased pain tolerance and a capability of suicidal behaviours (e.g., using lethal means) developed over time. Repeated provocative and painful experiences increase pain threshold and decrease fear of death; and more painful or provocative events confer greater capability [10,11,12]. Nevertheless other distal factors such as mental health disorders, physical health, personality characteristics, and genetic predisposition increase the individual risk of developing desire to suicide [12].

The current study aims to draw upon the IPT and utilises psychological autopsy (PA) information to create lifecharts in order to explore the pathways to suicide and suicidal process in Australian farmers.

## 2. Methods

### 2.1. Participants

Eighteen interviews were conducted with the next-of-kin (NOK) of male farmers who had lived and died in Queensland (QLD; *n* = 12) or New South Wales (NSW; *n* = 6). The objective was to have one close informant (NOK) for each suicide case. Informants for QLD farmer suicides were identified and recruited directly from the Queensland Suicide Register (QSR), a suicide mortality database managed by the Australian Institute for Suicide Research and Prevention (AISRAP). Informants had given consent to the Queensland Police Service (QPS) to be contacted for research purposes following the suicide death of their NOK. Inclusion criteria implied that the deceased was a farmer/farm worker who worked/lived on a farm; or was involved in seasonal work where the position had been maintained for longer than six months and employment was continual; or unemployed (i.e., involuntarily stopped active farming) or recently retired within last six months before death; and, death occurred after 2006 (in order to attenuate recall bias) [13]. The nominated NOK was contacted by the clinical interviewer (L.K.) through a letter that explained the aims of the study. Approximately two weeks after posting the letter, the clinical interviewer contacted the NOK by telephone inviting them to participate and arranged an interview. All participants completed a consent form. Interviews were conducted by the clinical interviewer between July and December 2014.

Informants for NSW farmer suicides were identified and recruited with the assistance of the State Coroner’s Court of New South Wales. Researchers reviewed all of the suicide death files for the category of “farmer” in the National Coronial Information System (NCIS) database. The NCIS is a national internet-based data storage and retrieval system for Australian coronial cases. The Australian Institute for Suicide Research and Prevention (AISRAP) has existing ethical clearance and permission to access NCIS data. NOK informants for New South Wales farmer suicides were identified by the NSW State Coroner’s office, and then sent a letter to families explaining the aim of the study with a consent form to be forwarded to the clinical interviewer if consenting to participate. Once the completed consent form was received, the clinical interviewer contacted the informant by telephone to arrange a time for the interview. Interviews were conducted by the clinical interviewer between September and December 2015.

### 2.2. Data Collection and Measures

This study involved psychological autopsy (PA) methodology based on semi-structured interviews with individuals who knew the deceased well. A PA study allows the reconstruction of events around the suicide [13,14]. Given the nature and complexity of the problem and the limited knowledge about male farmer suicide, the PA method has been applied in a limited number of international farmer suicide studies [15]. Clinical interviews were conducted with a close relative of the male farmer who died by suicide. The PA instrument of this study was used by members of the present research team on other large-scale studies [16]. The interview consisted of the following sections: an unstructured discussion of the events leading to the death of the farmer, demographics (age, marital status, children, living arrangement, rural/urban status, education, employment, income, residency status, ethnicity, religion); circumstances of death (method, location and suicide note); history of suicidal behaviour/ideation and exposure to suicidal behaviours; medical and psychiatric history. Scales included the Interview for Recent Life Events (IRLE) [17] a semi-structured method, covering a range of recent and past life events to create life charts; and the Mini-International Neuropsychiatric Interview (MINI) [17] to determine post-mortem diagnoses was administered to the NOK to complete about the deceased. The MINI [18] explores 17 disorders (e.g., depression, suicidality, and alcohol dependence) according to Diagnostic and Statistical Manual (DSM) [19] diagnostic criteria. Studies have demonstrated concordance of DSM diagnoses by informant’s interview [20].

A life chart template [21] was adapted from one that had been previously successfully used by members of the research team to identify life pathways in the LGB population [22]. The template is similar to that used by Fortune et al. [23] to analyse youth suicides in the UK. The life chart categories were based on those of the IRLE [17]. Life events related to farming (i.e., natural disaster) were included in both the interview and the life chart template. The template for the life chart interview included following categories: employment, education, financial events, romantic relationships, non-romantic relationships, bereavement (including object loss) and family health, physical health, legal, residence change (move-intra, inter-city and interstate), mental health (including alcohol and other drugs (AODs)) and suicidality, contact with health services, suicide exposure, natural disasters and other farming related.

### 2.3. Procedure

This project was approved by the Griffith University Human Research Ethics Committee (CSR/08/13/HREC). PA interviews were conducted by a trained clinical interviewer (L.K.) by telephone. The majority of informants were ex-spouses of the deceased (*n* = 8), followed by siblings (*n* = 5), parents (*n* = 4), and an in-law (*n* = 1) (*M_age_* = 63.4 years, *SD* = 7.8). Interviews were conducted in a confidential room and recorded. Interviews took place a minimum of six months after the death of the farmer. Informants did not receive compensation in exchange for participation.

The first author (L.K.) drafted life charts based on interviews, which were further reviewed by the second author (K.K.). Two authors (L.K. and K.K.) independently reviewed the life charts in order to cluster farmer suicide cases in a meaningful way and to identify similar patterns in the pathways. The researchers then met to discuss and come to an agreement. “Typical” life charts were created to provide a visual illustration of the representative features of the lives of the farmers in each group. That is, the most common life events for each group were placed into a chart in a characteristically occurring sequence. The researchers (L.K. and K.K.) then independently coded the life charts for the 18 cases as belonging to one of three groups. One case was carefully considered in detail, with consensus achieved with an agreed final coding (Kappa = 0.913). Three subgroups with pathways were defined by agreement.

## 3. Results

Farmers who died by suicide were aged 23–77 years (*M* = 53 years, *SD* = 13.4). The average ages at death for QLD and NSW farmers were 50.2 years (*SD* = 13.8) and 60.2 years (*SD* = 10.1), respectively (*t*(16) = −1.46, *p* = 0.163). Eight (44.4%) of these were between 55 and 64 years of age, five (28%) between 45 and 54 years, two were over 65 years, and one farmer was each aged between 15 and 24 years, 25 and 34 years and 35 and 44 years, respectively. The majority of farmers in the study lived/worked in QLD (72%, *n* = 13). Most farmers were found to have a diagnosis of a mental disorder at time of death (*n* = 17; 94% current depression). More than half used firearms, none of the seven men who died by hanging owned nor had access to a firearm previous to death. Characteristics of the study sample are presented in Table 1.

Life charts generated from PA interviews evidenced two pathways with different suicidal processes:

Group 1—situational (*n* = 14; 78%)—characterised by a brief period of interpersonal or work stressors, with an acute suicidal process and without direct communication of intent and/or deliberate self-harm to family and/or health professional;

Group 2—protracted (*n* = 4; 22%)—characterised by longstanding established mental health issues (i.e., established psychiatric disorder), with intermittent periods of hospitalisation and suicide exposure, with a protracted suicidal process with direct communication of intent to family and/or health professional.

**Figure 1:**
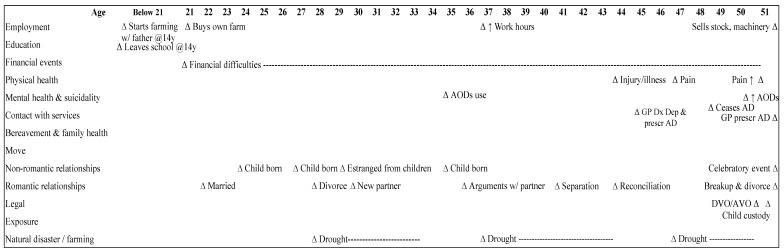
Typical life chart of Group 1.1—*Romantic relationship problems* (*n* = 9). AD = Antidepressants; AODs = Alcohol and other drugs; AVO = Apprehended Violence Order; DVO = Domestic Violence Order.

Within Group 1, two sub-groups were identified:

(1) Romantic relationship problems (*n* = 9; 50%): relationship breakdown featured in these cases: separation (*n* = 6; 67%) and divorce (*n* = 3; 33%) as shown in Figure 1. An acute stress response to situational factors, that is, relationship breakdown and child custody or paternity problems *(n* = 5; 56%) with a background of mental health problems (*n* = 9; 100%) and suicidality (*n* = 8; 89%), perpetuated by AODs (i.e., alcohol and/or cannabis) abuse/dependence (*n* = 7, 78%), were observed as prominent features. Interpersonal problems (i.e., work-related) (*n* = 5; 56%), were reportedly evident across the lifespan for this group, with three farmhands experiencing dismissal from a place of employment (33%) (Table 2).

Farmers in this subgroup were aged 30 to 58 years. Seven farmers (78%) had experienced previous/current AODs abuse/dependence, financial difficulties, and/or work problems over the course of their life. An undiagnosed mood disorder with psychotic features was a feature of three farmers (33%) in this subgroup. Four (44%) men had sought professional health treatment for their mental health in the two weeks prior to death (one in the last three months), or were in the care of a health professional with an appointment pending within the next week. None of these men were working at time of death, attributed to the adverse impact of mental illness.

**Figure 2:**
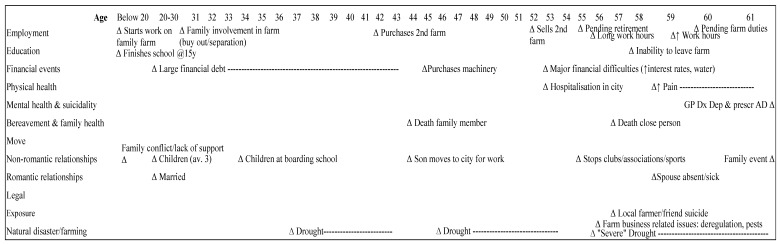
Typical life chart of Group 1.2—*Financial difficulties/pending retirement* (*n* = 5). AD = Antidepressants.

(2) Financial difficulties/pending retirement (*n* = 5; 28%): the main features at time of death were financial difficulties (*n* = 5; 100%) and pending retirement (*n* = 4; 80%) as shown in Figure 2. An acute stress response to situational factors, related to recent long work hours, pending farm duties as well as farm related issues experienced in the years previous to death, (e.g., closure of the mill, deregulation of milk, and crop disease) (Table 2).

Farmers in this subgroup were aged 52 to 62 years. The majority of farmers in that group (*n* = 4; 80%) were preparing financially for retirement. Exploration of typical life-chart for this group revealed that at time of death, all farmers in this subgroup had been diagnosed with depression and prescribed antidepressants in the weeks prior to death; however, reportedly had not previously experienced symptoms of, nor sought treatment for, depression. All men also appeared to be experiencing an anxiety disorder (e.g., generalized anxiety disorder (GAD), obsessive compulsive disorder (OCD) or social anxiety with panic), however, had not been formally diagnosed at the time of death. All, except one farmer, were in contact with a doctor, and two farmers were under the care of a psychiatrist in the previous 12 months before death and had been diagnosed with depression and anxiety and prescribed pharmacotherapy.

**Figure 3:**
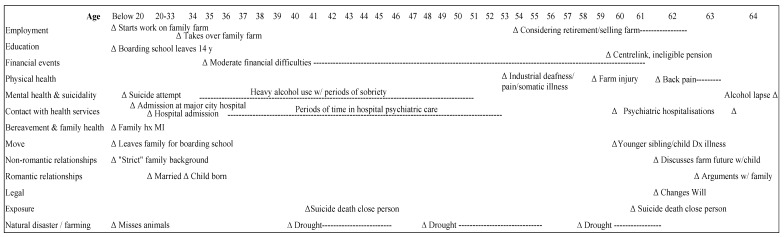
Typical life chart of Group 2—*Long-term mental health problems* (*n* = 4). MI = Mental Illness.

Group 2: Long-term mental health problems (*n* = 4; 22%): characterised by evidence of an established psychiatric disorder (*n* = 4; 100%), an additional shared feature was exposure to suicide on at least two occasions across the lifespan as shown in Figure 3. Suicidality was protracted over many years and men had contact with the mental health treatment during adulthood with three men verbally communicating intent to die by suicide previous to death.

Farmers in this group were aged 63–77 years (*n* = 3; 17%), with one young man in the 20–24 year age bracket (Table 2). Three were owners/farmers, and one a farmhand on his parents’ farm. The defining feature of this group was the presence of a long-standing diagnosed psychiatric disorder (bipolar disorder, schizophrenia, or depression). In addition, all men had experienced the suicide death of at least three close persons (i.e., family members) in their lifetime and the young man who died had experienced the suicide attempt of a close friend. All farmers in this group had sought professional treatment for their mental health (and suicidal ideation) in the two weeks prior to death, or were in the care of a health professional with an appointment pending within the next week or as an outpatient with a health professional.

## 4. Discussion

To the best of our knowledge, the current study is the first of its kind in the Australian or international contexts to analyse the pathways to suicide in male farmers. Findings revealed two distinct pathways: (a) “situational” where suicide has occurred in response to acute situational life stressors; and (b) “protracted” where suicide has been a protracted process—farmers experienced a diagnosed psychiatric disorder as a life stressor over many years.

Farmers who died as a result of situational stressors were observed to have had limited exposure to suicide and were more likely to not communicate intent. Two main groups of farmers with situational stressors were identified in the study. The first group was middle-aged and the predominant feature in their pathways was having relationship problems and breakup (separation and divorce). Recent relationship breakdown has been suggested as being central to understanding suicidal process in Australian males. The suicidal process identified, appears congruent with that identified by Kõlves et al. [24] who presented that recent separation from a partner entails a significant acute risk factors of subsequent suicidal behaviour, particularly for men. Separation and divorce have been shown to cause shame and anger, threatening masculinity and traditional gender roles and lead to acute stress, depression and substance abuse [25]. Furthermore, separation and family related conflict have been found to increase suicide risk in people with substance use disorders [26]. In addition, separation and divorce also impact the male role as a father, which may be limited or removed. In line with the IPT recent relationship breakup may lead to social isolation, especially in rural and remote areas and also to feeling of burden to family and friends [27].

The second subgroup of farmers with situational stressors were older males, characterised as experiencing financial difficulties across their lifespan and pending retirement preceded death. The suicidal process in this subgroup was congruent with that identified previously by other researchers [2,7], who observed work and financial concerns or difficulties linked to stress and mental illness, availability of firearms along with low rates of treatment and a lack of a confiding relationship were important factors for suicide. Depression was commonly reported at time of death. Current financial hardship (i.e., difficulty to paying bills, having to ask welfare for money, or inability to engage in activities) has been observed strongly associated with depression [28]. Important external factors impacting the financial situation of that group were drought and the Global Financial Crisis.

Farmers, who demonstrated a protracted suicidal process, had experienced severe psychiatric disorders and psychiatric hospitalisations since early adulthood and had frequent exposure to suicide. Mental illness is known as an important risk factor for suicide [29], however, previous farmer suicide studies have not focussed on the impact of long-term psychiatric disorder on male farmers. At time of death, the farmers reportedly were unable to work as a result of their health concerns. Recent evidence shows that psychiatric disorders that are symptomatically associated with thwarted belongingness and perceived burdensomeness [30]. Further, disorders with potential exposure to painful and provocative events (e.g., schizophrenia and bipolar disorder) are associated with increased acquired capability [30].

One frequently shared factor across all groups was physical illness and pain, particularly in older farmers. Overall, suicide in older adults has been associated with physical illness, functional impairment and other losses (e.g., bereavement) more than interpersonal relationships, financial and occupational problems [31,32]. However, it appears that older farmers due to the manual nature of their work are dependent upon their physical and mental health. Hence, these interrelated factors perhaps are more salient as antecedents to suicide. Physical decline and bereavement have been suggested as needing to be understood in terms of reflecting loss of social bonds and participation [12,33,34]. Previous PA studies have identified depression, functional impairment, pain, physical illness and social isolation as key risk factors that elevate risk for perceptions of burdensomeness [12,34]. Further, pain, independent of other risk factors, has been found significantly associated with perceived burdensomeness and predicting suicide ideation [35], while depression and social isolation are posited as elevating risk for thwarted belongingness [35]. Recent evidence suggests that depression increases the likelihood of an individual experiencing thwarted belongingness and/or perceived burdensomeness. In addition, disorders associated with both thwarted belongingness and perceived burdensomeness may place individuals at greatest risk if acquired capability develops [30].

By the IPT [10,11,12] suicidal desire will lead to suicidal behaviour in the presence of the acquired capability for suicide. The repeated exposure to painful or provocative events provokes an increased threshold of fear and pain insensitivity leading the individual towards habituation to fear and pain—increasing capability of suicide. Farmers in this study had ready access to, and familiarity with firearms, critical elements in determining the suicide method [31]. Farmers in this study had been familiarised with shooting firearms from a young age. During drought, farmers reportedly needed to euthanise animals. This type of life events, containing painful and provocative elements in conjunction with the interpersonal components of thwarted belongingness and perceived burdensomeness, have been suggested as resulting in a fearlessness of suicide whereby acquired capability has been habituated [32].

Individual pathways to suicide cannot be translated without wider social and environmental context. One of the important contributing environmental factors reflected also on the pathways of majority of suicide cases was drought. In Australia and North America, drought has been associated with increased health effects and risk of suicide in males [9,36]. In Australia, drought has led to an increased workload on farms, and women have sought off-farm employment to contribute financially [37]. During times of rural crisis, such as drought, farmers have been observed to feel powerless as they perceive to have a lack of control over factors that are linked to farming success [38]. Alston [37] observed that, although employment structures have changed, traditional gender roles have not. The reliance of spouses earning off-farm financial assistance has had a negative impact on the male sense of self [37]. Some researchers have argued that farmers, as business owners, work within a broader and transitional economic and political framework and factors that threaten the autonomy of farmers, are needing to be contextualised within a social, cultural, economic and political framework, not just at an individual mental health level [39]. Extraneous to interpersonal factors, it perhaps is these factors that are the indicators leading to perceived burdensomeness and thwarted belongingness—i.e., farmers perceive themselves as powerless against the political and economic framework of agriculture.

Findings from this study suggest that male farmers require targeted prevention, assessment and treatment strategies in their rural and regional communities across the lifespan from boarding school to planning retirement and succession planning. Strategies to consider could include: restricting access to means particularly on presentation to a health professional, relationship and family counselling, financial counselling particularly retirement preparation, public health and stigma reduction campaigns particularly around understanding the symptoms of depression and anxiety and the association between physical and mental health and mental illness. For example during the last drought, NSW implemented the Drought Mental Health Assistance Package (DMHAP) to assist affected farmers [4]. Mental health training was provided to frontline agencies, and workshops conducted, with the aim of reducing stigma and increase mental health knowledge within the community [4]. Subsequently, a Rural Mental Health Support Line was established. This program was extended as part of the NSW Health Rural Adversity Mental Health Program (RAMHP), which integrated general practice into the drought response and raising awareness about alcohol use [40]. Nevertheless, evaluations have been primarily based on immediate feedback [40]; evaluation on the efficacy to reduce suicide are needed. In addition to the community related activities there is need also for national policies, which could provide financial advice and support dignified financial exit, especially for older farmers, but also support training and new businesses [37].

There are limitations that should be acknowledged. As a psychological autopsy study, it is subject to recall bias, potentially providing a skewed understanding of their loved one’s thoughts, feelings and behaviours leading to their death by suicide [13]. Nevertheless, validity studies comparing PA diagnoses with clinicians treating the individuals before death has been tested [20,41]. Study findings may be unique to the relatively few farmers included in this study; hence, it is may not be generalizable to all farmers and needs to be replicated with a larger sample, including more young men from the younger age group and farm labourers who have been found to have higher suicide rates [38]. Although a greater number of Queensland informants were recruited compared to New South Wales, findings may be representative of these samples given that Queensland has been found to have twice the rate of farmer suicide compared with New South Wales [42].

## 5. Conclusions

To our knowledge, this is the first study to complement the PA method with the life charts approach in order to understand the pathways to suicide in Australian farmers. This study observed differences in the suicidal process. For most farmers, intent was not communicated, nor was there previous suicide exposure, instead the process was acute, in response to two salient situational stressors of romantic relationship breakdown for middle aged men, and for older men, financial difficulties pending retirement. For other farmers, who experienced many years of a psychiatric disorder, the process was protracted. As the IPT by Joiner [11] suggests, there a number of interrelated factors that are to be investigated in order to predict and prevent suicide.

## Figures and Tables

**Table 1:** Characteristics of the study sample (*n* = 18).

Characteristics	*n*	%	Characteristics	*n*	%
*Employment*			*Marital Status*		
Owner/manager	11	61.1	Single/never married	3	16.7
Manager/Senior	1	5.6	Married	6	33.3
Farmhand/stationhand	6	33.3	Separated	5	27.8
			Divorced	4	22.2
*Occupation length in years*			*Living arrangements*		
2–3	1	5.6	Alone	8	44.4
4–5	2	11.1	With spouse/partner and Children	3	16.7
6–9					
10–15	1		With spouse/partner	4	22.2
>15	14		With parents	3	16.7
*ARIA* ^1^			*Education*		
Inner regional	5	27.8	<Grade 10	7	38.9
Outer regional	11	61.1	Grade 10	5	27.8
Remote	2	11.1	Grade 12	2	11.1
			TAFE	3	16.7
			University	1	5.6
*Commodity*			*Income*		
Mixed crop and livestock	3	16.66	<$25,000	9	50.0
Beef cattle	5	27.78	$25,001–$60,000	5	27.8
Dairy	1	5.55	$60,001–$95,000	2	11.1
Sugar cane	1	5.55	$95,001–$120,000	-	
Sheep	2	11.11	>$120,000	1	5.6
Fruit or nut grower	1	5.55	Unknown	1	5.6
Mixed livestock	2	11.11			
Other	2	11.11			
*Size of farm (hectares)*			*Suicide method*		
<50	3	16.7	Hanging	7	38.9
51–500	1	5.6	Firearms	11	61.2
501–5000	10	55.6			
5001–499,000	1	5.6			
>500,000	1	5.6			
Unknown	2	11.1			

^1^ Accessibility and Remoteness Index of Australia.

**Table 2:** Groups by defining and principle features of life charts and other characteristics of male farmers who died by suicide (*n* = 18).

Group	Situational (Acute)		Long-Term (Protracted)	
Sub-Group	Romantic Relationship Problems (*n* = 9)	Financial Difficulties/Pending Retirement (*n* = 5)	Long-Term Mental Health Problems (*n* = 4)	
**Age**	30–58 years old	63–77 years old	52–62 years old	20–24 years old
**State**	**QLD** (6), **NSW** (3)	**QLD** (4), **NSW** (1)	**QLD** (2), **NSW** (1)	**QLD** (1)
**Employment**	Farmer/owner (4), farmhand (4), caretaker (1)	Farmer/owner (5)	Farmer/owner (3)	Farmhand
**Sub-group defining features**	**Separation** (6)	**Financial difficulties** (5)	**Unipolar depression** (3)	Schizophrenia
	**Divorce** (3)	**Pending retirement** (4)	**Bipolar disorder** (1)	Unipolar depression
	**Relationship problems** (9)		**Dementia** (1)	
**Further specifiers**	**Mental health problems** (9)	**Long work hours** (3)	**Suicide exposure** (3)	**Suicide exposure**
	**Suicidality** (8) **Alcohol/drugs (previous and current abuse/dependence)** (7)	**Impact of Global Financial Crisis** (GFC) (2)		
**Other characteristics**	Financial issues (7)	Bereavement and family health (5)	Physical health (3)	Suicidality
	Natural disaster (7)	Mental health (5)	Natural disaster (3)	Parental conflict
	Work problems (7)	Natural disaster (5)	Bereavement and family health	Legal
	Physical health (6)	Physical health (4)	(3)	Bereavement and family health
	Child custody/paternity issues/estrangement (5)	Pending farm duties (3)		Non-romantic relationships
	Legal (4)	Lifetime parental conflict (3)		
	Anniversary/celebratory occasion (4)	Anniversary/celebratory occasion (e.g., birthday/Christmas) (2)		
	Bereavement and family health (4)	Legal (2)		
	Family hx of mental illness (4)	Family farm (2)		
	Suicide exposure (3)	Seasonal issue/farm disease (2)		
	Period of unemployment (3)			
	Non-romantic relationships (3)			
	Domestic Violence Order or Apprehended Violence Order (2)			
	Lifetime parental conflict (2)			
	Seasonal issues (2)			
	Family farm (2)			
**Psychiatric diagnoses (MINI)**	Suicidality current (8)	Unipolar depression current (5)	Unipolar depression current (1)	Suicidality current
	Unipolar depression current (8)	Unipolar depression past (3)	Unipolar depression past (3)	Unipolar Depression
	Unipolar depression past (5)	Suicidality current (4)	Suicidality current (3)	Psychotic Disorder
	Alcohol abuse current (4)	GAD Current (3)	Bipolar disorder current (2)	
	Substance dependence current (4)	Bipolar Disorder past (2)	Family Hx bipolar disorder (1)	
	Family Hx Bipolar Disorder (4)	Family Hx Bipolar disorder (1)	GAD current (1)	
	Psychotic disorder current (3)	PTSD lifetime (2)	Alcohol abuse current (1)	
	GAD current (2)	Social anxiety current (1)	Alcohol dependence current (1)	
	Alcohol dependence current (2)	OCD current (1)	Alcohol dependence past (1)	
	Alcohol dependence past (2)	Antisocial Personality disorder	Agoraphobia current (1)	
	PTSD lifetime (2)	(1)	Agoraphobia past (2)	
	Antisocial Personality disorder (2)		Social phobia current (2)	
	Bipolar disorder current (1)		OCD current (1)	
	Bipolar disorder past (1)		Panic disorder current (1)	
	PTSD current (1)		Panic disorder lifetime (2)	
	Conduct disorder (1)			

Psychiatric diagnoses and other defining characteristics in bold are a feature of the entire (sub) group.
